# A Minimal Optical Trapping and Imaging Microscopy System

**DOI:** 10.1371/journal.pone.0057383

**Published:** 2013-02-25

**Authors:** Carmen Noemí Hernández Candia, Sara Tafoya Martínez, Braulio Gutiérrez-Medina

**Affiliations:** 1 Program in Molecular Biology, Instituto Potosino de Investigación Científica y Tecnológica, San Luis Potosí, Mexico; 2 Program in Physics, Universidad Autónoma de San Luis Potosí, San Luis Potosí, Mexico; 3 Advanced Materials Division, Instituto Potosino de Investigación Científica y Tecnológica, San Luis Potosí, Mexico; University of Cambridge, United Kingdom

## Abstract

We report the construction and testing of a simple and versatile optical trapping apparatus, suitable for visualizing individual microtubules (∼25 nm in diameter) and performing single-molecule studies, using a minimal set of components. This design is based on a conventional, inverted microscope, operating under plain bright field illumination. A single laser beam enables standard optical trapping and the measurement of molecular displacements and forces, whereas digital image processing affords real-time sample visualization with reduced noise and enhanced contrast. We have tested our trapping and imaging instrument by measuring the persistence length of individual double-stranded DNA molecules, and by following the stepping of single kinesin motor proteins along clearly imaged microtubules. The approach presented here provides a straightforward alternative for studies of biomaterials and individual biomolecules.

## Introduction

Over the last two decades, optical traps (or optical tweezers) have become a standard tool in the physical and biological sciences, allowing the measurement of sub-nanometer displacements of optically trapped microparticles, as well as the exertion of piconewton-level, controlled forces on these particles [1], [2], [3]. Taking advantage of this versatility, studies on the mechanical and biochemical properties of biomolecules at the single-molecule level using optical tweezers are now widespread. In these assays, individual molecules, attached to trapped particles, can be pulled on or stretched (or even twisted) using the laser trap, and the molecular displacement and force (or torque) responses can be measured with high spatial (∼1 nm) and temporal (∼100 kHz) resolution [4]. These experiments provide unique information on molecular mechanisms that complement traditional biochemical studies.

Typically, an optical tweezers arrangement consists of a single laser beam tightly focused by a microscope objective of high numerical aperture (NA) (1.0–1.4), which traps microparticles near its focal point [5], [6]. Position detection of the trapped particle relative to the laser beam axis is achieved through back focal plane (BFP) detection, where the trapping beam (or a secondary beam of low-power) is directed to a position-sensitive detector (PSD) located in a plane conjugate to the condenser BFP [7]. In many trapping setups, these requirements are implemented by substantial modifications of a commercial inverted light microscope in order to accommodate holders, mounts, and a stable stage platform fitted with a piezoelectric stage (for fine sample movement). Alternatively, an optical trap can be built entirely from individual optical components, providing increased flexibility in the design, often reducing costs, and facilitating the choice of parts. This last option is the one we have followed in this work.

Successful development of an optical tweezers apparatus often requires appropriate means to visualize small objects (∼20–200 nm). Indeed, a variety of cellular and single-molecule assays require the localization and subsequent tracking of nanoparticles [8], or the imaging of individual slender polymer filaments such as microtubules (MTs, with diameters of only ∼25 nm) [9]. Because small objects scatter light weakly, their visualization has traditionally not been performed using bright field microscopy. Instead, localization is achieved by employing a number of specialized imaging techniques, in the case of MTs: fluorescence [10], dark field [11], polarization [12], phase contrast [11], or Nomarski differential interference-contrast (DIC) microscopy [13]. However, some of these visualization methods exhibit inherent limitations in the context of optical trapping. For example, it is well-known that the Wollaston prisms required in DIC optics can introduce significant asymmetries in the optical trap [14]. Likewise, because the Wollaston prisms generally produce or recombine two displaced beams with orthogonal polarizations, DIC microscopy prohibits the operation of an optical tweezers setup where polarization of the trapping beam requires high purity and independent adjustment (as in an “optical torque wrench” [15]). Introducing optical elements such as prisms, apertures, phase masks, or polarizers in the laser beam path unavoidably constrains the possibilities for optical trapping, especially when control of phase or polarization effects is necessary, as in holographic [16] or interferometric [17] optical tweezers.

Recently, it has been shown that unlabeled, individual MTs can be visualized by computer-enhanced bright field microscopy (CEBFM) [18], a method that does not require the addition of specific optical elements or processing instrumentation to effectively remove the background, reduce noise, and enhance contrast of images. CEBFM provides an alternative of remarkable simplicity to visualize small objects, and is therefore ideally suited to complement optical trapping. In bright field microscopy, ample access to optical paths in the setup is ensured, and it is possible to independently optimize Koehler illumination and the BFP detection stage of the optical trap, allowing excellent imaging and trapping. By using CEBFM, a basic microscope could be fit to perform standard single-molecule “gliding filament” or optical trapping “bead” assays without resorting to advanced imaging modalities. Furthermore, CEBFM may prove useful in emerging microscopy techniques that use image contrast to perform biophysical measurements and that incorporate optical tweezers as a tool for microparticle manipulation, such as “defocusing microscopy” [19] (see Section “Digital image processing”).

In this work, we describe the design, construction, and testing of an elementary optical tweezers instrument that allows trapping and visualization of weakly scattering objects, integrating a straightforward approach for the study of biomaterials and individual biomolecules. Our design is based on CEBFM together with a single laser beam coupled into the microscope for optical trapping and BFP detection. This arrangement keeps the number of mechanical and optical components to a minimum, resulting in ease of construction and cost-effectiveness that do not compromise final stability or resolution. To test the capabilities of our system, we performed two single-molecule assays and compared our results with previous reports. We have measured the persistence length of individual double-stranded DNA (dsDNA) molecules at relatively high ionic strength ([Na^+^] = 150 mM), and have observed the stepping of single kinesin motor proteins as they advance along clearly imaged, individual MTs. To our knowledge, this is the first time that the classic cytoskeletal motor protein bead-assay has been demonstrated using plain bright field illumination to localize filament substrates.

## Results and Discussion

### Optical and Mechanical Layout

**Figure 1:**
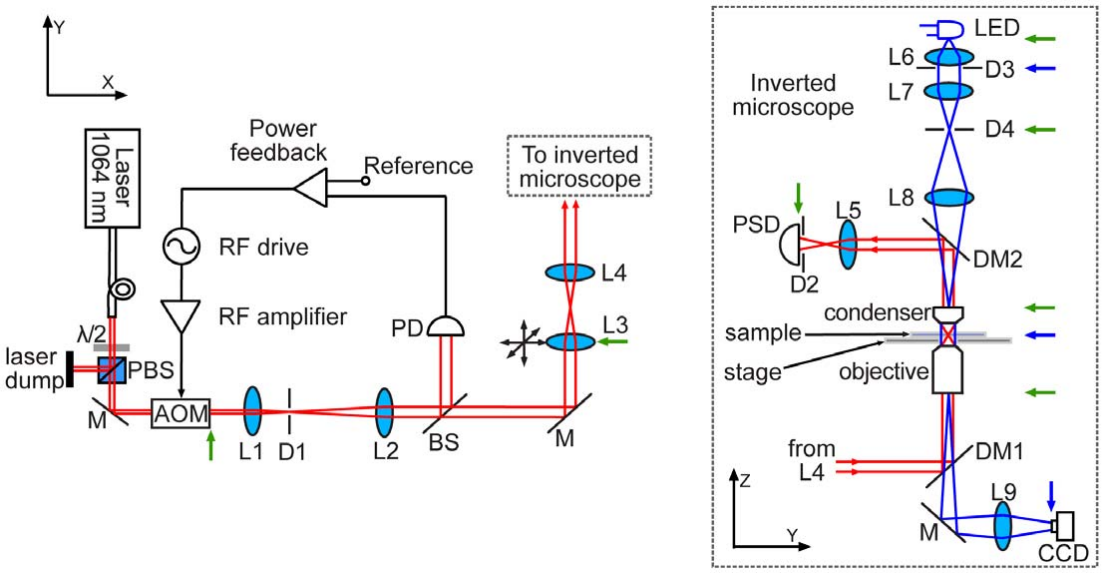
Schematic of the optical tweezers instrument. A fiber-coupled laser provides the trapping light (red lines), the intensity of which is controlled with a combination of a λ/2-waveplate and a polarizing beam splitter (PBS). An acousto-optic modulator (AOM) is part of a feedback loop that stabilizes laser intensity. The trapping beam is coupled into and out of a custom-built inverted microscope (black dotted line box) using two dichroic mirrors (DM). Illumination (blue lines) for the inverted microscope is provided by a blue LED. The green and blue arrows indicate the positions of the two sets of optically conjugated planes that satisfy the conditions for Koehler illumination and back focal plane detection. See text for details. BS: beam sampler, PD: photodiode, M: mirror, L: lens, D: diaphragm iris, PSD: position-sensitive detector.

Our optical tweezers system (shown in Figure 1) was built entirely from individual components, mounted on a vibration-isolation optical table (I-2000, Newport). Trapping light is provided by a CW, Yb-doped, fiber laser (YLR-10-1064-LP, 10 W, λ = 1064 nm, IPG Photonics) featuring excellent pointing stability (deviations <1 µrad/s), and good power stability (fluctuations ∼2%). A 10 m-long optical fiber coupled to the laser allows installation of the laser head (together with the noisy power electronics) in a separate room. The laser is nominally operated at ∼4.2 W of output power, above the minimum ∼3.0 W, that according to the manufacturer accounts for the stability figures cited above.

We now describe our setup in detail. The end terminal of the laser fiber is secured onto the optical table, and a combination of a half-wave plate and a polarizing cube beamsplitter (PBS) control the laser power going to the optical trap. To provide means of automatically steering the laser trap on the sample plane, an *x*-*y* acousto-optic modulator (AOM) (DTD-274HD6M, IntraAction) is placed immediately after the PBS, and an iris diaphragm (D1) selects the (+1,+1) diffracted beam that emerges from the AOM. Next, two lenses (L1 and L2), forming a Keplerian telescope, expand the beam to a final waist size, *w,* of ∼3 mm, corresponding to a beam diameter that roughly matches the diameter of the objective back aperture (∼6 mm). Two additional lenses (L3 and L4) then form a 1∶1 telescope, with L3 (see Figure 1) mounted to an *x-y*-*z* translation stage to provide fine manual control to steer and collimate the laser beam going into the objective. A stage-mounted lens is a useful feature during the initial alignment of the trap. To transform beam deflection by either the AOM or lens L3 into beam translation in the specimen plane and minimize beam clipping, the AOM and the lens L3 are optically conjugate to the back aperture of the objective [6] (see Figure 1).

The AOM is useful not only to steer the trap but also to control the laser power going into the objective. Our instrument is placed in a room that does not have temperature control, and we have found that the output laser power may drift during the course of the day by as much as ∼5%, presumably due to room temperature changes. To correct for this problem, a laser power feedback loop was implemented using the AOM. A beam sampler placed after the AOM picks up part of the main beam and sends it to a photodiode, whose signal is amplified and fed into a data-acquisition board (U12, LabJack) connected to a computer. A proportional-integral-derivative (PID) feedback routine is implemented in software, using LabView 8.5 (National Instruments), and its corrected amplitude signal is communicated to the AOM driver (AFG3022B, Tektronix) via a USB port. Finally, the *x* and *y* outputs of the AOM driver are each amplified by an RF amplifier (ZHL-3A, Minicircuits), and connected to the AOM, closing the feedback loop. The resulting feedback control has a characteristic time response of ∼1 s, and maintains the power constant (to a level of ∼0.2%) for hours. Alternatives to this feedback scheme using an AOM driver are available [20].

To afford imaging of the specimen, trapping chamber manipulation, and laser focusing by the objective, an inverted microscope was built. Because one of the main purposes of this work is to attain visualization of weak light-scattering objects by means of bright field microscopy (without the aid of additional optical parts or special types of illumination), the components of the optical train of our microscope are arranged to satisfy Koehler illumination. Among other advantages of Koehler illumination, the field is homogeneously bright, image contrast is optimum, and maximal lateral and axial resolutions are achieved [21].

In our microscopy system (see Figure 1), the illuminator is composed of a high-power light-emitting diode (LED) (LXHL-LR5C, 700 mW, peak λ = 455 nm, Luxeon) as the light source, together with a collector lens (L6) and a field iris diaphragm (D3) placed immediately adjacent to L6. The LED is driven by an inexpensive analog current controller, originally designed for laser diodes, that provides extremely stable current (∼10^−4^ rms variations) up to 500 mA (design available at: http://george.ph.utexas.edu/~meyrath/informal/laser%20diode.pdf). This intensity stability is necessary to perform appropriate background subtraction during image processing (as described in the next section). The collector lens collimates incoming light from the LED, and an additional lens (L7) focuses the image of the light source onto the condenser iris diaphragm (D4). To maximize contrast during imaging, we close this aperture almost entirely (∼85%) to effectively produce a point-like illumination source. Relay lens (L8) images the condenser iris onto the condenser BFP.

The condenser lens (1.4NA, Zeiss) is mounted on an *x*-*y*-*z* translation stage (461-XYZ-M, Newport), and aligned to focus an image of the field diaphragm onto the sample plane. This ensures that the specimen and the images of the light source are in different (reciprocally related) planes, and that the specimen is illuminated with a set of plane waves. Following the optical train, the objective lens captures the illuminating plane waves and creates an image of the light source onto the rear focal plane of the objective (exit pupil). We use an infinity-corrected, oil-immersion objective of high numerical aperture (100X/1.3NA/PlanFluorite, part RMS100X-PFO, Olympus). Finally, a tube lens (L9) captures illuminating and scattered light from the specimen and images the sample onto an inexpensive CCD camera (STC-TB33USB-B, Sentech) that is connected to the computer via a USB interface. With the condition for Kohler illumination satisfied in this optical arrangement, two mutually reciprocal sets of optically conjugated planes are formed. The first set includes the field diaphragm, the sample plane, and the camera sensor plane. The second set includes the light source, the condenser iris, the rear focal plane of the condenser, and the back focal plane of the objective.

Coupling of the trapping laser into and out of the microscope is achieved using two dichroic mirrors (Z1064RDC-SP, Chroma). To perform BFP detection, a single lens (L5) images the rear focal plane of the condenser onto a PSD (DL100-7-PCBA2, Pacific Silicon Sensor). A diaphragm (D2) placed directly in front of the PSD modulates the NA of the detection optics, optimizing the PSD detection signal [22]. The output voltage signals from the PSD (fitted with a built-in preamplifier) are further amplified using a custom-made electronic circuit (200 kHz bandwidth) and sent to a data-acquisition board (PCI-6052E, National Instruments) fitted in a computer, where data are processed by custom software written in LabView. A laser power of 90 mW applied to the trap (measured before entering the objective rear pupil) was typically used for our experiments. To block the trapping laser whenever necessary, an inexpensive, custom-made mechanical shutter was used (design available at: http://george.ph.utexas.edu/~meyrath/informal/shutter.pdf).

An advantage of our optical trapping and microscopy setup over previous designs is that BFP detection and Koehler illumination are effectively decoupled and can be optimized independently. In typical microscopes working under Koehler illumination, the condenser iris is located at the back focal plane of the condenser lens (entrance pupil). This arrangement is not ideal for an optical tweezers arrangement, as any aperture placed in the proximity of the condenser compromises BFP detection. Conversely, although standard optical trapping systems often do not use the condenser iris, here it is necessary to maximize contrast during visualization of weak light-scattering specimens. We resolved this potential conflict by using relay lens L8 to image the condenser iris onto the condenser BFP, leaving the condenser lens entrance pupil unobstructed. Lens L8 thus allows us to adjust the condenser iris aperture without affecting the NA of the BFP detection optics.

The mechanical design of the microscope is completed with a piezoelectric stage (Nano-LP100, Mad City Labs) that is used for fine sample movement, and a supporting crossed-roller-bearing mechanical stage (Manual MicroStage, Mad City Labs) that provides coarse positioning. The *x*-*y*-*z* piezoelectric stage has nanometer-level step resolution over 100×100×100 µm. The stacked stages are supported by four 8″-tall, 1.5″-diameter stainless steel posts that provide mechanical stability to the stage platform. All of the condenser, detection, and illumination optics are mounted on a breadboard that is suspended vertically on a structure formed by two large vertical construction rails (XT95, Thorlabs), cross-linked at the top by a third rail, and further supported by one additional beam joining the top rail to the optical table.

### Digital Image Processing

Images are digitally processed to remove background, perform frame averaging, and enhance contrast. We implemented computer-based image processing essentially as described in Ref. [18], with appropriate improvements for our work. All of the procedures described here are implemented in software using LabView 8.5 (add-on package Vision, National Instruments).

First, using the piezoelectric stage, the surface of the coverslip is brought into focus and subsequently defocused by a few micrometers. At this point, the sample is oscillated in the *x* or *y* direction (about 5 micrometers in amplitude, 2 seconds in period) using the piezoelectric stage, while background images (typically 250) are simultaneously acquired. An average of the background images provides a reference image that is subsequently subtracted from all incoming frames. As background frames are acquired this way, images belonging to any relatively strong scattering objects that may be stuck to the coverslip surface and present in the field of view are defocused and blurred in the final reference background image, thereby minimizing their influence in subsequent processing. This allows for changes in the field of view without the need to acquire a new background reference. After completing this operation, focus is readjusted to visualize the sample.

**Figure 2:**
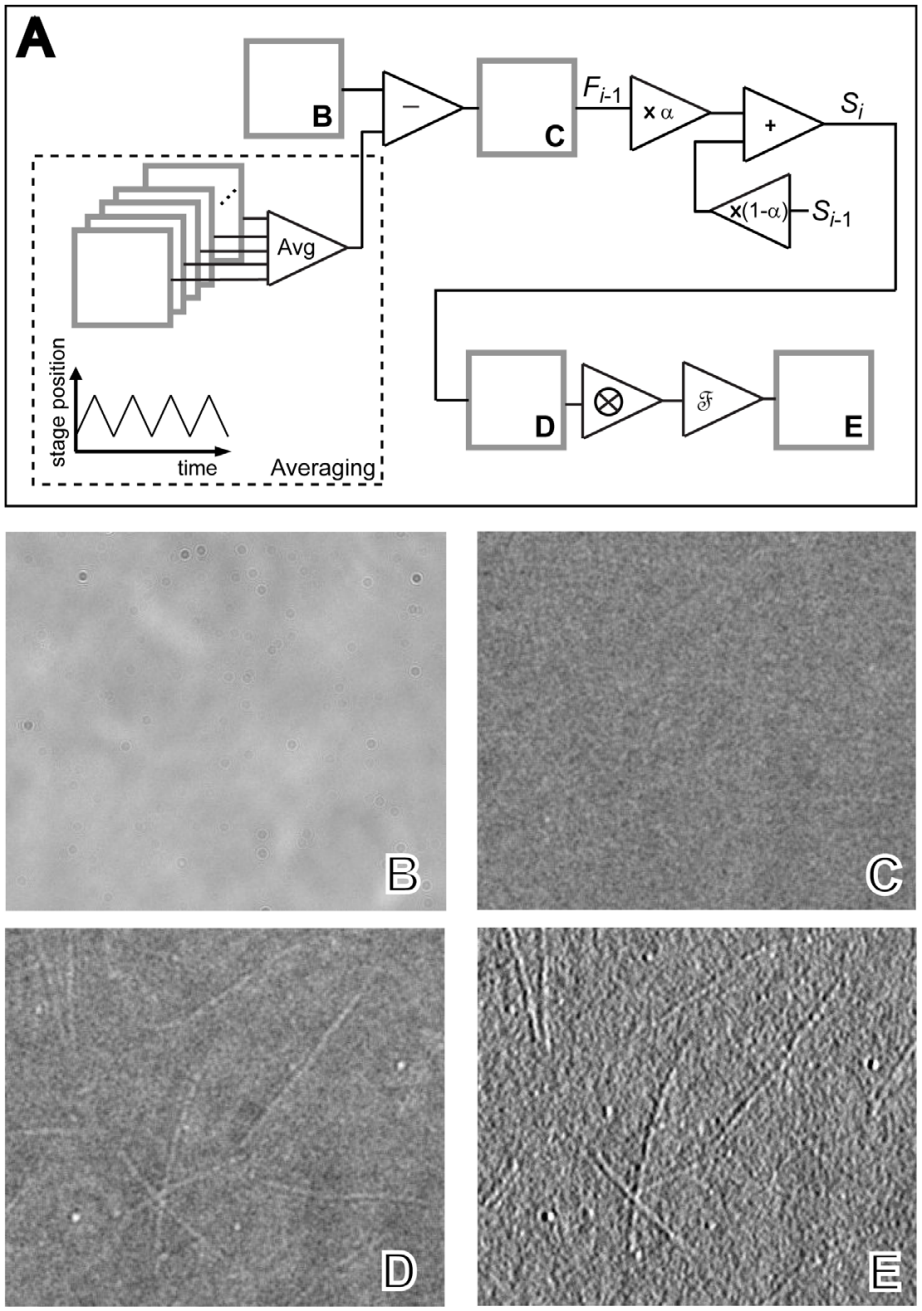
Individual microtubules are visualized using bright field microscopy together with digital image processing. (A) Flow diagram of digital image processing, depicting operations for background acquisition (dashed area) and subtraction, exponential averaging, and contrast enhancement. (B-E) Frames show MTs immobilized on coverslips, as imaged during the various stages of processing marked in (A): (B) raw image; (C) the same as (B) with the background removed; (D) the same as (C) after multiple-frame exponential averaging; (E) the same as (D) after a convolution routine (shown with the symbol 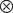 in the diagram) that enhances contrast in the vertical direction, and a fast-Fourier-transform routine (shown with the symbol 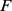 in the diagram) that smoothes out rapidly-varying spatial features. Field of view is 24 µm × 21 µm.

Next, background-free images are averaged using a simple exponential-averaging (or exponential-smoothing) procedure, during which incoming time series data are averaged using exponentially decreasing weights [23]. One advantage of exponential over boxcar averaging is that it is easier to implement, as only two data (the last, smoothed datum and the incoming datum) need to remain in memory. In addition, the weight assigned to recent or older data can be readily adjusted. Thus, let *F_i_* represent the *i* ^th^ original, background-free image acquired sequentially in time and *S_i_* the corresponding final, smoothed image. Exponential averaging is implemented according to the following rule [24]:
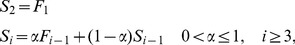
(1)where parameter α is the smoothing constant (see Figure 2A). Full or zero weight is assigned to the most recent datum in the time series by setting α = 1 or α = 0, respectively. We typically use α = 0.3 in our experiments.

Background-free, exponentially-averaged images can be further processed using two additional routines that enhance contrast and reduce noise: (*i*) a spatial convolution routine, which involves a point-by-point multiplication of the pixel values of the original image multiplied by the corresponding pixel values of a filtering convolution mask [18], and (*ii*) a fast Fourier transform smoothing routine, which removes spatial features within a given size range.

**Figure 3:**
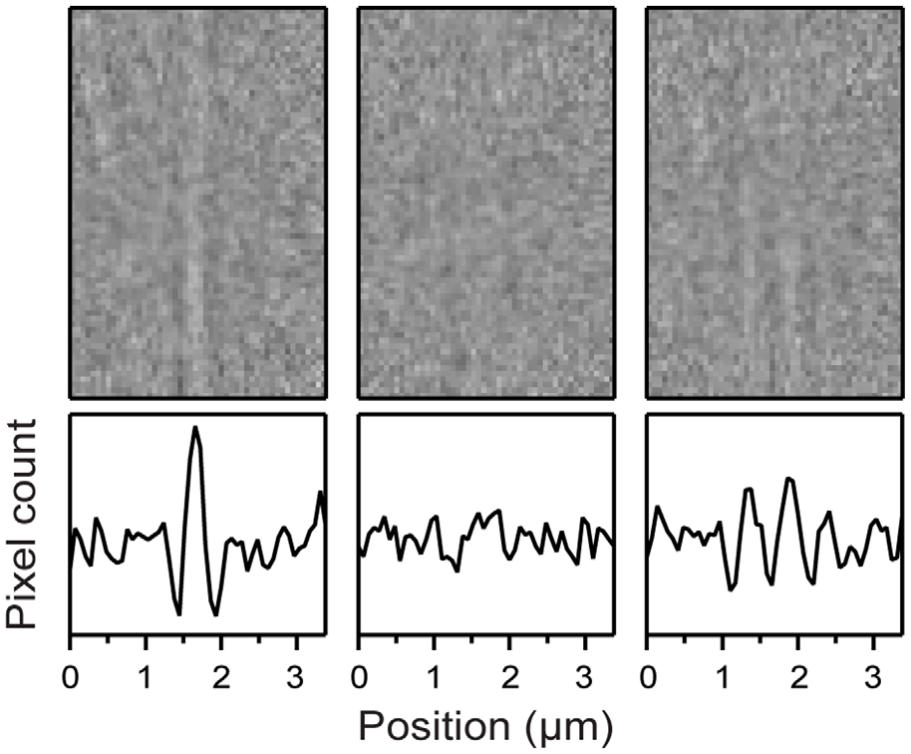
Microtubule image contrast is optimized by microscope defocusing. Images of a single MT (top row) displaying the same field of view when the focus is placed 300 nm below (left), precisely at (center), and 300 nm above (right) the coverslip surface. The bottom row shows the corresponding pixel count profiles, obtained for each image by averaging all the pixel count values along a given pixel column. Images were digitally-processed as described in the text to remove background and perform exponential averaging (α = 0.3). No convolution or fast-Fourier-transform routines were applied in this case. Field of view is 3.4 µm × 4.8 µm.

To test our system, we visualized MTs, polymer filaments that are involved in cellular structure and organization [25], previously immobilized on coverslips (see Materials and Methods). Figures 2B–2E show that individual MTs are clearly distinguished, with an apparent thickness determined by the diffraction limit of the microscope (∼250 nm). We noticed that the contrast of MT images changes as the coverslip surface is brought in and out of focus, reaching an optimum when the coverslip surface is slightly (∼300 nm) defocused (see Figure 3). This fact is consistent with the early observation by Fritz Zernike that when a bright field microscope is focused precisely, the image of a thin, transparent specimen disappears due to interference of light diffracted by the specimen and of background illumination, and contrast is maximized only by slightly defocusing the microscope [21]. Recently, this effect has been used to develop “defocusing microscopy,” a quantitative visualization method where image contrast (shown proportional to the amount of defocusing and to the curvature of the specimen) is measured, from where it is possible to learn about the material properties of the sample [19]. In our experiment, the MT filaments are transparent under visible light and present enough shape curvature, therefore they act as effective phase objects despite being hollow and having a diameter of only λ/20.

The previous MT imaging result demonstrates that our setup achieves excellent sample visualization at standard video rates (∼30 Hz), offering several advantages over a recent system that also imaged MTs using bright field microscopy [18], among which are: improved background subtraction (by using a light source with excellent intensity stability and by moving the piezoelectric stage during background acquisition), real-time specimen visualization (by implementing exponential averaging of images), and the possibility of using the full NA of the condenser (by using relay lens L8 to image the condenser iris onto the condenser BFP). Most importantly, the instrument presented here has the full capabilities of optical trapping for measuring molecular-level displacements and forces.

### Calibration of the Optical Trap

**Figure 4:**
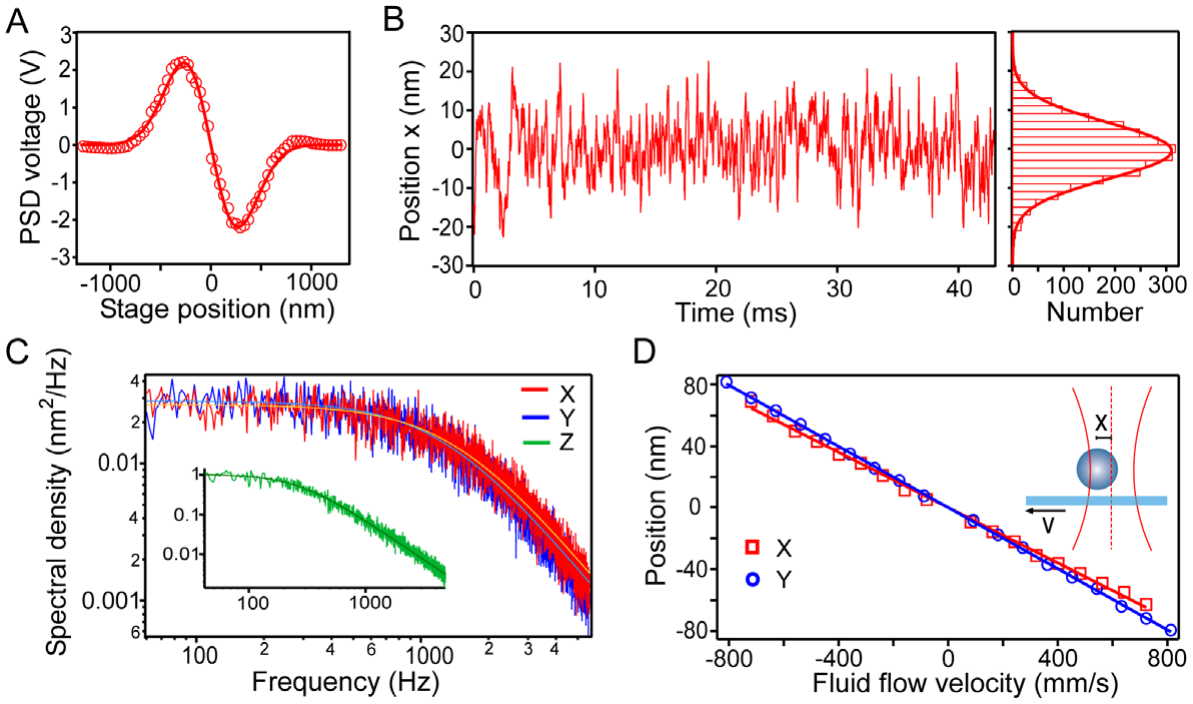
Calibration of the optical trap. (A) Response of the position-sensitive detector (PSD) as a bead immobilized on the coverslip is scanned across the laser beam along the *x* axis (red circles). The data are well-fit by the derivative of a Gaussian (red line), from which the nanometer to volt conversion factor is determined, ξ*_x_* = 0.013 V·nm^−1^. (B) The position of a trapped bead along the *x* axis is monitored and the respective histogram is well-fit by a Gaussian (thick red line). The position variance is 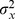 = 51 nm^2^ (or, the raw PSD signal variance is 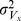 = 0.0086 V^2^). Using the principle of equipartition of energy, the trap stiffness is found: κ*_x_* = 0.080 pN·nm^−1^. (C) The power spectrum of the position signal along the *x* (red line), *y* (blue line), and *z* (green line, inset) directions is shown, together with fittings to a Lorentzian function for *x*, *y*, and *z* (red, blue, and green thin lines, respectively). Measurement of the roll-off frequency of the fit yields the trap stiffness: κ*_x_* = 0.063 pN·nm^−1^ and κ*_y_* = 0.055 pN·nm^−1^. (D) In the Stokes drag calibration method, a trapped bead is subject to fluid drag by moving the stage at velocity *v*, resulting in the bead moving to a new equilibrium position that is measured (inset). Bead displacement vs. stage velocity data are shown for the *x* (red squares) and *y* (blue circles) axes. Linear fits to the data (red and blue lines) yield the slopes: *s_x_* = 0.090 nm·µm^−1^·s, and *s_y_* = 0.099 nm·µm^−1^·s (or the corresponding raw-signal slopes *s_Vx_* = 0.0012 V·µm^−1^·s, and *s_Vy_* = 0.0013 V·µm^−1^·s), which yield trap stiffness along each direction: κ*_x_* = 0.076 pN·nm^−1^, and κ*_y_* = 0.063 pN·nm^−1^. As discussed in the text, ξ and κ can alternatively be found using: ξ = 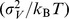
(γ/*s_V_*), and κ = γξ/*s_V_*. This method yields ξ*_x_* = 0.012 V·nm^−1^, and κ*_x_ = *0.07 pN·nm^−1^ for the example presented here. The average value of κ*_x_* using all four of these methods provides the best estimate of trap stiffness along *x*: κ*_x_* = 0.072±0.007 (mean ± SD). Calibrations were performed using 0.54-µm diameter polystyrene beads.

We perform position and force calibrations using well-established procedures for trapping spherical beads [6]. First, the relationship between the PSD voltage in a given direction, *V_x_*, and the displacement of the bead from the equilibrium position, *x*, is determined by scanning a bead affixed to the coverslip surface across the laser beam. As shown in Figure 4A, the resulting profile *V*(*x*) is well-described by the derivative of a Gaussian function, and the slope of the central, linear part of the profile is the conversion factor from nanometers to volts, ξ*_x_* (units of V/nm), which is subsequently used to convert all PSD signals along *x* from volts to nanometers. Next, the stiffness of the optical trap, κ, along the lateral directions is established by using three separate methods, which are based on measurements of particle position variance, power spectrum, and Stokes’ drag, respectively. The stiffness along the axial direction is established using the particle position variance and power spectrum methods only.

Briefly, for the first method, the variance of the position signal of a trapped bead (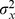) is measured and, using the principle of equipartition of energy, the stiffness κ*_x_* is given by κ*_x_* = 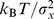, where *k*_B_ is Boltzmann’s constant and *T* is temperature (see Figure 4B). For the other two procedures, the drag coefficient (γ) of a bead of radius *r* immersed in a fluid of viscosity η, γ = 6πη*r*, is assumed to be known. In the second method, the power spectrum of the position signal of a trapped bead is computed and fitted by a Lorentzian profile (see Figure 4C). From a measurement of the roll-off frequency of the fit, *f*_0_, the stiffness can be derived as κ = 2πγ*f*_0_. Finally, in the third method, a steady drag force is exerted on a trapped bead by moving the sample with the piezoelectric stage at constant speed (*v_x_*). The resultant viscous drag places the bead at a new equilibrium position, *x* = γ*v_x_/*κ*_x_* (from Stokes’ law), that is measured. This procedure is then repeated for various *v_x_*, and the stiffness is inversely proportional to the slope of the linear fit of the *x* vs. *v_x_* data (see Figure 4D).

Alternatively, the variance and the Stokes’ drag methods can be combined to provide simultaneous position (ξ) and force (κ) calibrations along the lateral directions, using the raw PSD signal, *V_x,y_*. The advantage of this approach over the previous independent methods is that it does not require an explicit position calibration (based on coverslip-stuck beads), and can therefore be applied to free beads. In this case, we measure the variance of the position signal, 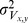 = *k*_B_ *T*ξ*_x,y_* ^2^/κ*_x,y_* (units of V^2^), and the slope of the *V_x,y_* vs. *v_x,y_* linear fit in the Stokes’ drag method, *s_x,y_* = γξ*_x,y_*/κ*_x,y_* (units of V·µm^−1^·s). These two relationships are combined to yield ξ*_x,y_* = 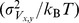
(γ/*s_x,y_*), and κ*_x,y_* = γξ*_x,y_*/*s_x,y_*. Like the power spectrum and Stokes’ drag methods, with the previous procedure it is assumed that the drag coefficient of the bead is known. For a sphere near a surface, the relationship γ = 6πη*r* for the hydrodynamic drag no longer holds, due to surface proximity effects [6]. Accordingly, to determine the drag coefficient, we take into account Faxen’s law in all our measurements. Very good agreement is obtained between all calibration methods presented here, resulting in variations of ∼10% in values of κ*_x,y_*.

### The Persistence Length of dsDNA

**Figure 5:**
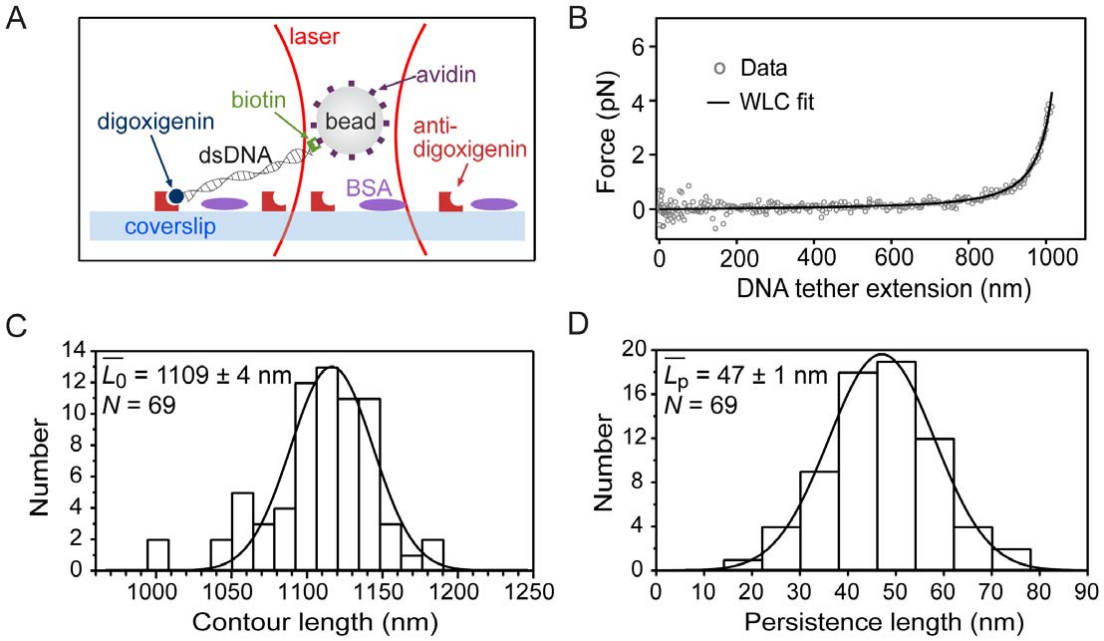
Stretching single dsDNA molecules. (A) Schematic of the surface-based assay, not to scale. (B) A typical force-extension record (black circles) together with a worm-like chain (WLC) polymer model fit (black line). (C) Histogram of contour length values. Fit to a Gaussian (black line) yields: 1116±2 nm (μ ± σ_μ_). (D) Histogram of persistence length values. Fit to a Gaussian (black line) yields: 47.0±0.2 nm (μ ± σ_μ_). Legends display data mean ± SEM.

A number of studies have measured the persistence length of dsDNA under a variety of buffer conditions [26], [27], [28], [29]. We tested our optical trapping instrument by pulling on individual dsDNA molecules, using a standard surface-based assay [28], at a relatively high ionic strength ([Na^+^] = 150 mM). Following established protocols, a ∼3.1 kb dsDNA template was attached by the 5′ end of one of its strands to a 0.7 µm-diameter polystyrene bead, and affixed by the 5′ end of the complementary strand to the coverslip surface (see Figure 5A and Materials and Methods). The procedure to measure force-extension curves for dsDNA is described in Ref. [27].

Briefly, surface-tethered beads were held by the laser tweezers and centered with respect to the surface attachment point, along the *x* and *y* directions, by adjusting the piezoelectric stage position such that stage motions in the ±*x* or ±*y* directions produced symmetric bead displacement responses (as measured by the PSD). Next, beads were placed at a specified distance (20 nm) above the coverslip surface, taking into account the effective focal shift (∼0.8) of the optical trap [6]. Then, the DNA molecule was stretched by moving the coverslip horizontally (using the piezoelectric stage) in steps of 10 nm in distance, 10 ms in duration. For each step, *x*,*y*,*z* bead displacements were recorded at a rate of 10 kHz and averaged in each direction. Finally, the extension of the elongated molecule (*L*) was determined from the known stage movement and the measured bead position with respect to the trap, and the force (*F*) acting on the molecule along the stretching direction was determined from the position of the bead with respect to the trap and the trap stiffness [27]. Because the method used here to determine bead-surface distances is precise to 20 nm [14], the distance between the bead and the surface was set to 0 nm to obtain force-extension curves. All DNA molecules were stretched in the ±*x* and ±*y* directions. To discriminate single from multiple tethers, only ±*x* and ±*y* symmetrical force-extension curves were considered for analysis. We report results from stretching along the *y* direction only. For each molecule, data corresponding to the +*y* scan direction were superimposed on the –*y* scan direction, and treated as a single *y* record. Care was taken to perform measurements within the respective linear regions of position and force calibrations.

Figure 5B shows a typical single-molecule force-extension record. Maximal stretching forces of 4 pN were applied to all molecules. In this low-force regime, the elasticity of DNA is essentially entropic, and the corresponding *F* vs. *L* relationship is well-described by the worm-like chain (WLC) polymer model [30]:
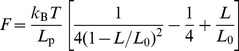
(2)where *L*_0_ and *L*_p_ are the contour length and the persistence length of the polymer, respectively. Force-extension records were well-fit using the WLC model (see Figure 5B), from which values for *L*_0_ and *L*_p_ were inferred. Statistical analysis of data for *L*_0_ and *L*_p_ (see Figs. 5C and 5D) yielded measured values: 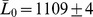 nm and 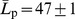 nm (mean ± SEM), which are in excellent agreement with the expected value for the contour length *L*_0_ ∼1053 nm (see Materials and Methods) and with previous reports of *L*_p_ at comparable ionic strengths [29].

### The Stepping of Kinesin along MTs

Kinesin is a homodimeric motor protein that is involved in intracellular transport, using the energy of ATP hydrolysis to ferry cargo along MTs [31]. Previous studies using optical trapping, among other single-molecule techniques, have determined that kinesin advances in 8.2-nm increments and can sustain retarding loads as large as ∼7 pN before stalling [32]. In traditional optical-tweezers based bead assays of kinesin, DIC microscopy is used to localize MTs [9]. Here, we demonstrate the versatility of our instrument by observing kinesin motors stepping along MTs, where we simultaneously use both optical trapping and the excellent visualization features available from our experimental setup.

**Figure 6:**
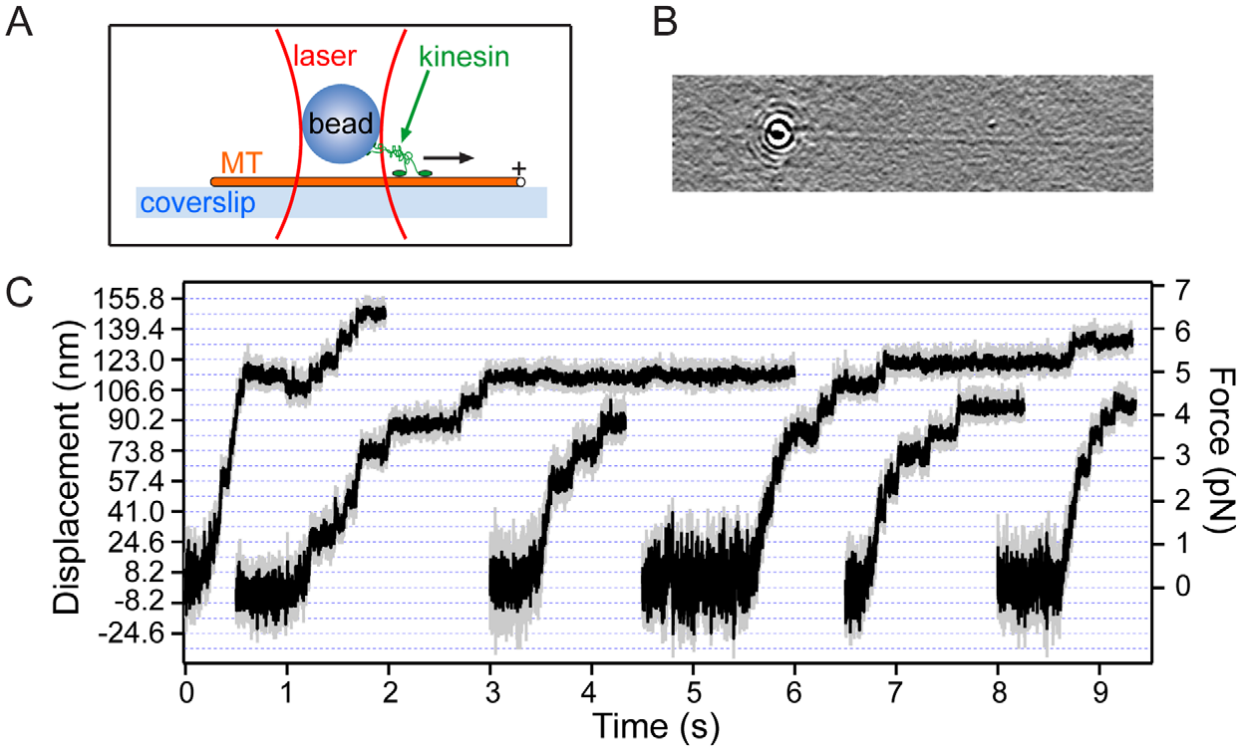
Kinesin stepping along MTs. (A) Schematic of the kinesin bead assay, not to scale. (B) A kinesin-carrying trapped bead is placed directly above an individual MT, visualized using digital image processing. Field of view is 27 µm × 6 µm. (B) Representative records of kinesin-driven bead motion (gray line: unfiltered trace acquired at 20 kHz, black line: trace after a 20-point median filtering), display steps at 8.2-nm intervals (dashed horizontal lines) and the development of forces up to ∼7 pN. [ATP] = 100 µM.

To record kinesin motion, a standard motility bead assay was carried out (see Figure 6A) [33]. Single recombinant kinesin motors were attached to 0.54-µm polystyrene beads (see Materials and Methods). Kinesin-carrying beads were optically trapped and placed directly above individual MTs that were immobilized on the coverslip surface, previously identified using the microscope’s digitally-enhanced imaging (see Figure 6B). When kinesin binds to the MT in the presence of ATP in the buffer solution, it advances unidirectionally towards the ‘plus’ end of the MT. In our experiment, the (fixed) optical trap was used both to measure the stepping of kinesin, and to exert a retarding force on motors that increases as beads are transported away from the center of the laser beam. For MTs aligned along the *x* direction, the retarding force on kinesin is determined through Hookés law (*F_x_* = -κ*_x_*·*x*). Figure 6C presents examples of kinesin stepping records obtained in this work, showing that expected steps of 8.2 nm are clearly resolved. Additionally, we observed maximal forces sustained by motors before stalling or detachment from the microtubule of ∼7 pN, consistent with previous, detailed studies of kinesin motility [32].

### Conclusions

An optical trapping system has been developed with exceptional visualization features at minimal cost and complexity. The technique of CEBFM provides an alternative to elaborate microscopy techniques for high-contrast visualization, avoiding the inclusion of optical elements in the instrument (such as prisms, apertures or polarizers) that could compromise operation of optical trapping. Using our instrument, it is possible to achieve independent control of BFP detection (in optical trapping) and Koehler illumination (in sample visualization). The approach presented here is uniquely suited for single-molecule experiments and studies of biomaterials at the micro- and nano-scales. Unconventional optical trapping techniques, such as an optical torque wrench (for rotating microparticles), or holographic and interferometric optical tweezers (for creating multiple-trap arrangements) can be incorporated immediately without sacrificing imaging capabilities. The setup can also be readily expanded to incorporate additional features, such as a second trapping beam (to perform off the surface, multiple-bead assays) [34], or strategies to maintain a constant force during experiments (a ‘force clamp’) [14], [35].

## Materials and Methods

Unless specified, all reagents were purchased from Sigma. Microscope slides and coverslips were cleaned prior to use for 5 min in a plasma cleaner (Harrick Plasma) at 1 Torr (ambient air). Flow channels were made using double-sided tape as described [36]. All buffers were filtered before use through 0.22 µm filters (Millipore).

### Preparation of dsDNA

A 3039-bp dsDNA coding for a fragment of the *blr-1* gene of *Trichoderma atroviride* [37] was amplified by a polymerase chain reaction (PCR) (Applied Biosystems). To allow attachment of the dsDNA to a polystyrene bead and to the coverslip, two functionalized PCR primers (Integrated DNA Technologies) were used: a forward primer (biotin-5′-GGGCTTCTACCAGACAAACCA-3′), and a reverse primer (digoxigenin-5′-CGCTCTTCTCGTATTGAAGCC-3′). The reaction tube contained: 5.0 µL of the 5XPCR buffer (Promega), 2.5 µL of 25-mM MgCl_2_ (Promega), 0.5 µL of 100-mM dNTPs, 0.5 µL of 10-µM reverse primer, 0.5 µL of 10-µM forward primer, 1.0 µL of cDNA from the *blr-1* gene (generous gift of Sergio Casas Flores, IPICYT, Mexico), 0.5 µL of Taq Polimerase (GoTaq, Promega), diluted in 15 µL of Milli-Q water. The PCR ran for 25 cycles with an alignment temperature of 58°C and 1 min for extension. The amplified fragment was purified using a QIAQuick PCR purification kit (Qiagen). The expected length of the dsDNA molecule is ∼1053 nm, computed by taking into account a 3039-bp chain with 0.34 nm rise per bp [38], together with ∼20 nm for the biotin-streptavidin and digoxigenin-anti-digoxigenin linkages.

### Recombinant Kinesin

We expressed the homodimeric, recombinant kinesin construct DmK401, a His-tagged derivative of *Drosophila melanogaster* kinesin heavy chain, that includes the first 401 N-terminal residues (previously described [39]). Briefly, BL21(DE3) cells transformed with plasmid pCA1 (generous gift of Steven Block, Stanford University) were grown to logarithmic phase in Luria Broth medium (10 g/L tryotone, 5 g/L yeast extract, 10 g/L NaCl) supplemented with 0.1 mg/mL ampicillin (GIBCO). Kinesin expression was chemically induced with 1 mM IPTG (Invitrogen) at 27°C during 12 h. Cells were lysed by sonication in extraction buffer (200 mM Na_2_HPO_4_, 50 mM NaCl, 2 mM imidazole, 20 uM ATP, 2 mM MgCl_2_, 1 mM DTT, 1 mM phenylmethylsulfonyl fluoride (PMSF), and protease inhibitor cocktail (P8465, Sigma); 1% Tween was added after sonication). Cellular lysate was clarified by centrifugation (30 min, 15,000 rpm, 4°C). Clarified lysate was stored at −20°C in 10% glycerol, and used in motility assays.

### Single-molecule Assays

#### dsDNA flexibility

A solution of 0.05 mg/mL of antidigoxigenin (3210–0488, Spherotech) in phosphate buffer saline (137 mM NaCl, 2.7 mM KCl, 10 mM Na_2_HPO_4_, 2 mM KH_2_PO_4_) was introduced in a flow channel, and incubated for 10 min. After washing with 200 µL of washing buffer (5 mg/mL BSA, 77.4 mM Na_2_HPO_4_, 0.1% Tween), the channel was filled with a sample of 0.25 nM dsDNA diluted in phosphate buffer (77.4 mM Na_2_HPO_4_), and incubated for 10 min. Unbound DNA was removed by flowing 200 µL of washing buffer through the channel. Finally, 30 µL of 730-nm diameter, avidin-coated beads (generous gift of Steven Block, Stanford University), diluted in phosphate buffer to a final concentration of ∼1 pM, were introduced into the channel, and the flow cell was sealed using nail polish.

#### Kinesin motility assay

The motility, bead assay follows previous experiments. Briefly, 10 µL of the stock solution of 540-nm diameter, streptavidin-coated beads (Spherotech, SVP-05–10) were diluted in 70 µL of PEMBSA buffer (4 mg/mL BSA, 80 mM PIPES, 1 mM EDTA, 4 mM MgCl_2_, pH 6.9), and sonicated for 10 min., after which 20 µL of penta-His biotin conjugate antibody (34440, Qiagen) were added. After incubating for 1 hour at room temperature, beads were washed 5 times by centrifugation, and stored at 4°C. To bind kinesin to the beads, antibody-coated beads were diluted in assay buffer (3 mg/mL BSA, 0.05 M potassium acetate, 100 µM ATP, 1 µM DTT, 80 mM PIPES, 1 mM EDTA, 4 mM MgCl_2_, 0.02 mM Taxol (TXD01, Cytoskeleton), pH 6.9), sonicated, and incubated for 12 h at 4°C with clarified lysate diluted in assay buffer at various concentrations.

To immobilize MTs on coverslips, flow channels were prepared using coverslips coated with poly-L-lysine. A rack of plasma-cleaned coverslips was submerged in a solution of 600 µL of poly-L-lysine diluted with 300 mL of ethanol, incubated for 15 min, dried in an oven at 40°C, and stored in a closed container. Tubulin (TL238-C, Cytoskeleton) was polymerized to produce MTs as described [18]. Stabilized MTs were diluted in PEMTAX buffer (0.02 mM Taxol, 80 mM PIPES, 1 mM EDTA, 4 mM MgCl_2_, pH 6.9), introduced into the flow channel and incubated for 10 min. Unbound MTs were removed by washing the channel with 40 µL of PEMTAX. This procedure was followed to obtain samples used to tests the imaging quality of our microscope. To complete the kinesin motility assay, the channel was washed first with 60 µL of 20-mg/mL BSA diluted in PEMTAX (to minimize the sticking of beads to the coverslip surface), and then with 100 µL of assay buffer. Finally, 40 µL of kinesin-bead complexes were introduced into the channel and the flow cell was sealed. To minimize the presence of reactive oxygen species, final samples were protected with an oxygen scavenger system (0.25 mg/mL glucose oxidase, 0.03 mg/mL catalase, 4.7 mg/mL beta-D-glucose (MP Biomedicals)). To ensure motility in the single-molecule regime, kinesin dilutions were used in which only ∼50% of the tested beads in a given sample displayed movement. Data processing and analysis were performed using Igor Pro 5.0 (Wavemetrics).
